# Improvement of Selectivity of RALEX-CM Membranes via Modification by Ceria with a Functionalized Surface

**DOI:** 10.3390/polym15030647

**Published:** 2023-01-27

**Authors:** Irina Stenina, Polina Yurova, Aslan Achoh, Victor Zabolotsky, Liang Wu, Andrey Yaroslavtsev

**Affiliations:** 1 Kurnakov Institute of General and Inorganic Chemistry RAS, Leninsky Prospect 31, 119991 Moscow, Russia; 2 Faculty of Chemistry and High Technologies, Kuban State University, 350040 Krasnodar, Russia; 3 School of Chemistry and Material Science, University of Science and Technology of China, Hefei 230026, China

**Keywords:** RALEX, heterogeneous membrane, composite, ceria, surface modification conductivity, permselectivity

## Abstract

Ion exchange membranes are widely used for water treatment and ion separation by electrodialysis. One of the ways to increase the efficiency of industrial membranes is their modification with various dopants. To improve the membrane permselectivity, a simple strategy of the membrane surface modification was proposed. Heterogeneous RALEX-CM membranes were surface-modified by ceria with a phosphate-functionalized surface. Despite a decrease in ionic conductivity of the prepared composite membranes, their cation transport numbers slightly increase. Moreover, the modified membranes show a threefold increase in Ca^2+^/Na^+^ permselectivity (from 2.1 to 6.1) at low current densities.

## 1. Introduction

Electromembrane processes, in particular, electrodialysis, are widely used in various industrial processes for the purification and separation of substances in aqueous solutions, including wastewater, feed source demineralization, seawater desalination, production of organic acids and bases, etc., providing high purity products and the efficiency of ion separation [1,2,3,4,5]. Electrodialysis involves ion transport through ion-exchange membranes under an applied electric potential gradient [6,7,8]. The efficiency of electrodialysis, like most other electromembrane processes, depends on two of the main parameters of membranes: Their ionic conductivity and selectivity [9,10,11,12]. Ionic conductivity of cation exchange membranes is determined by the transport of counterions in a thin Debye layer near membrane pore walls with negatively charged sulfonic acid groups, while the non-selective transport of co-ions is determined by their transport through an electrically neutral solution localized in membrane pore centers [13,14,15]. Relatively cheap heterogeneous cation exchange membranes generally used in electrodialysis represent laminated mixtures of ion exchange resins (generally sulfonated copolymers of styrene and divinylbenzene), an inert binder, and reinforcing fibers introduced to increase the mechanical strength of membranes. However, the selectivity of transport processes in these membranes is generally low due to the formation of an additional system of large pores between grains of membrane-forming components [16]. The membrane surface also played an important role, since its heterogeneity causes electroconvection, which can significantly increase the mass transfer rate and promote ion mixing [17].

In recent years, considerable attention has been devoted to the extraction of valuable components from natural water and wastewater [18]. For example, the problems with separation of lithium and cobalt [19,20], lithium and magnesium [21,22] are well known. There are problems of separation of sodium and calcium [23]. In this regard, the search for ways to separate singly and doubly charged ions is attracting more and more attention [24,25,26]. A number of approaches have been proposed to improve the selectivity of industrial membranes [27], e.g., membrane profiling [28], deposition of a layer of oppositely charged polyelectrolyte or several layers of alternately charged polyelectrolytes [25,29,30].

Another way to improve transport processes in ion-exchange membranes is to introduce inorganic particles (dopants) into membrane matrices [31]. One of the promising dopants is ceria. Ceria-doped membranes are highly stable during fuel cell operation [32,33], effective in water purification by reverse osmosis [34] and nanofiltration, and even have antibacterial properties [35], which can favorably affect the stability and service life of membranes. The reason for the unique antioxidant and antibacterial properties of ceria is its ability to interact with highly reactive oxygen radicals (hydroxyl radicals, superoxide radicals, etc.) by reversible transition between the Ce^4+^ and Ce^3+^ states [36,37]. This can be very important for preventing the degradation (fouling) of membranes used in electromembrane processes [38,39].

Ceria-based materials are widely used in water treatment, which was described in detail elsewhere [40]. The ability of cerium phosphate to increase the monovalent permselectivity of anion-exchange membranes is also known [41]. From this point of view, it seems interesting to modify membranes with ceria, the surface of which contains phosphate anions. However, to the best of our knowledge, there are no reports in the literature on the use of membranes modified by surface-functionalized ceria in water treatment by electrodialysis. In the case of ceria-based materials, it is especially attractive to modify only the membrane surface with them for several reasons. Modification with ceria usually leads to a decrease in the membrane conductivity [42,43,44]. However, if only the membrane surface is modified, this effect would be less pronounced. Moreover, smaller amounts of reagents are needed for modification. Finally, modification of the membrane surface, as noted above, in some cases results in an enhancement of the selective transport of singly charged ions [27].

The purpose of the present work is to understand whether it is possible to change the selectivity of ion transport (e.g., Ca^2+^/Na^+^ permselectivity) by modifying only the surface of a heterogeneous membrane with surface-modified cerium oxide. A simple procedure of manufacturing of composite ion-exchange membranes with improved permselectivity was proposed. Novel composite RALEX-CM membranes asymmetrically modified with PO_4_-functionalized ceria were prepared and their transport properties as well as Ca^2+^/Na^+^ permselectivity were studied.

## 2. Materials and Methods

Sodium chloride (NaCl), hydrochloric acid (HCl), phosphoric acid (H_3_PO_4_), sodium phosphate monobasic (NaH_2_PO_4_), calcium chloride (CaCl_2_) were purchased from Chimmed (Moscow, Russia). Cerium(III) nitrate hexahydrate (Ce(NO_3_)_3_^.^6H_2_O) was purchased form Sigma-Aldrich (St. Louis, MO, USA). Deionized water was used throughout all experiments.

RALEX-solutions leads to an increase in the water uptake of the R_Ce_HPM membranes based on a copolymer of sulfonated styrene and divinylbenzene (Mega a.s., Straz pod Ralskem, Czech Republic) were preliminarily conditioned according to the standard procedure [45] in aqueous NaCl solutions of various concentrations (decreasing from 2 M to 0.01 M) to let membranes swell and in 5% HCl aqueous solution to convert membranes into H^+^-form. For asymmetric modification (on only one side of a membrane) by in situ method, conditioned RALEX-CM membranes were placed for 10 min in a two-compartment cell, one compartment of which was filled with 0.01 M Ce(NO_3_)_3_ solution, and the other was filled with deionized water. Then, cerium nitrate solution was replaced with 8% aqueous ammonia solution and the resulting system was kept under constant stirring in both compartments for 2 h. The notation R_Ce was used to refer to these modified membranes. To prepare materials with PO_4_-functionalized ceria, the R_Ce membranes were treated with 1 M H_3_PO_4_ for 1 h or 1 M NaH_2_PO_4_ for 24 h also on one side. The notations R_Ce_HP and R_Ce_NaP were used to refer to these modified membranes.

To convert membranes into the H^+^, Na^+^, or Ca^2+^-forms, membranes were kept for 2 h in 5% HCl, 0.1 M NaCl, or 0.5 M CaCl_2_ solutions, respectively. Then, membranes were washed with deionized water.

X-ray diffraction (XRD) patterns were collected using a Rigaku D/MAX 2200 (Tokyo, Japan) on CuKα radiation in the 2Θ range of 10-65° at 25 °C and processed using Rigaku Application software. The attenuated total reflection FTIR spectra were recorded using a Nicolet iS5 FTIR spectrometer (Thermo Fisher Scientific, Waltham, MA, USA) with a Specac Quest attachment over the range 4000–500 cm^-1^. The imaging of the membrane cross sections were performed by scanning electron microscopy (SEM, Tescan Amber, Prague, Czech Republic) and energy dispersive spectroscopy (Oxford Instruments, Abingdon-on-Thames, UK). Static contact angles of water on membranes were determined using a KRUSS DSA25 instrument by the optical water drop method. The volume of a water drop was 10 µL. Before measurements, membranes were kept at 30% relative humidity for 24 h. 

The water uptake (ω(H_2_O)) of membranes was measured on a TG209F1 thermobalance (Netzsch-Gerätebau GmbH, Selb, Germany) in the temperature range of 20–200 °C and calculated according to Equation (1):

ω(H_2_O) = (W_wet_ − W_dry_)/W_dry_ × 100%, (1)

where W_wet_ and W_dry_ represent the membrane weights before and after drying at 200 °C, respectively. The dopant content was determined as W_res_/W_dry_, where W_res_ represents the weight of a residue after membrane heating at 800 °C in air for 10 min. The dopant content determination is complicated by the fact that it is impossible to completely convert membranes from the Na^+^- to the H^+^-forms (remove all residual Na^+^ ions), and when a polymeric part of membranes is burned off, residual Na^+^ ions interact with the membrane sulfonic acid groups to form Na_2_SO_4_. At the same time, since the amounts of residual sodium ions in the pristine RALEX-CM and the modified membranes are different, it impossible to calculate the dopant content by subtracting W_res_ of the RALEX-CM membrane from W_res_ of the modified membranes. In addition, when the membranes are kept in HCl solution for conversion to the H^+^-form, ceria (dopant) partially dissolves, which also makes it difficult to correctly determine its amount in the membrane. The dopant content is estimated to be in the range of 3–7 wt %. For comparison, the dopant content of heterogeneous membranes bulk-modified with zirconia was much higher (up to 20 wt %) [46]. Ion exchange capacity (IEC) was determined by direct titration [45] of the membranes in the H^+^-form. IEC is given per 1 g of swollen membrane

The conductivity (σ) of the prepared membranes in Na^+^- and Ca^2+^-forms was studied using an Elins Z1500 PRO impedance meter (Elins LLC, Chernogolovka, Russia) in the frequency range of 10–1.5 × 10^6^ Hz in a potentiostatic mode with amplitude of 80 mV. To measure the conductivity, membranes were fixed between graphite electrodes in a Plexiglas cell. The measurements were carried out in deionized water in the temperature range 25–80 °C. The resistance (1/σ) at each temperature was found by extrapolating the impedance plots to the Z’ axis (the axis of active resistances).

The current-voltage (CV) characteristics were studied using a rotating membrane disk setup that allows setting the diffusion layer thickness (δ) by changing the rotation rate of the membrane disk in accordance with the Levich theory [47]:

δ = 1.61D^1/3^ν^1/6^ω^−1/2^, (2)

where D represents the electrolyte diffusion coefficient, ν represents the solution kinematic viscosity, and ω represents the angular velocity of the membrane disk rotation. The CV characteristics were recorded in the galvanostatic mode at the membrane disk rotation rates of 50–300 rpm in the mixed solution of CaCl_2_ and NaCl. The solution supply rate into the cathodic chamber was 7.5 ± 0.1 mL/min. The total concentration of CaCl_2_ and NaCl was maintained constant and equal to 0.03 mol-eq/L (c_NaCl_ and c_CaCl2_ was 0.015 and 0.0075 mol-eq/L, respectively). The concentrations of Ca^2+^ and Na^+^ ions were measured by liquid chromatography (Stayer, Aquilon, Podolsk, Russia). The Hittorf transport numbers (t_+_) were determined by Equation (3):

t_+_ = (c – c_0_)VF/I, (3)

where c_0_ and c represent the initial electrolyte concentration and the electrolyte concentration at the cathodic chamber outlet, respectively, V represents the volumetric rate of the solution supply, I represents the current, and F represents the Faraday constant. The Ca^2+^/Na^+^ permselectivity was calculated using Equation (4):

P_Ca/Na_ = t_Ca_c_0Na_/(t_Na_c_0Ca_) (4)

where t_Ca_ and t_Na_ represent the effective transport numbers, c_0Ca_ and c_0Na_ represent the concentration of NaCl and CaCl_2_ solutions, respectively.

## 3. Results and Discussion

**Figure 1:**
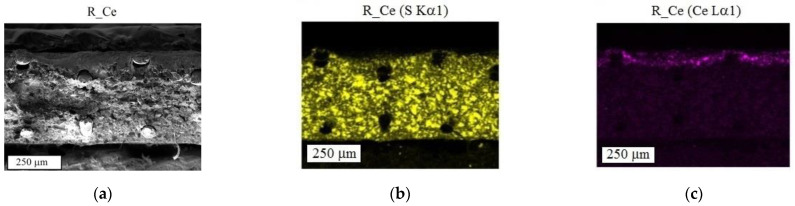

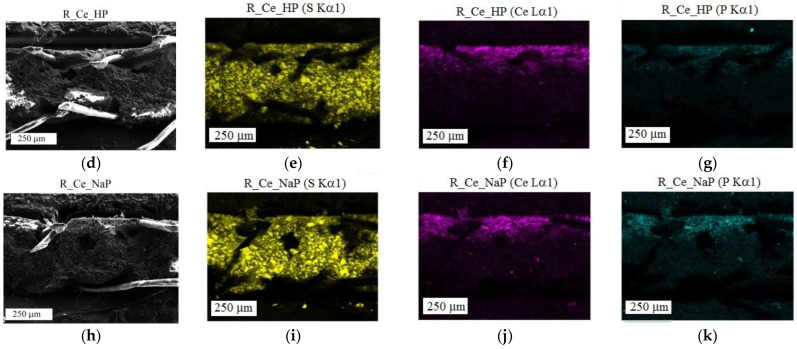
SEM images (**a**,**d**,**h**) and mappings of sulfur (**b**,**e**,**i**), cerium (**c**,**f**,**j**) and phosphorus (**g**,**k**) across the thickness of the R_Ce (**a**–**c**), R_Ce_HP (**d**–**g**) and R_Ce_NaP (**h**–**k**) membranes.

Figure 1 shows SEM images of the cross section of the modified membranes and the mappings of sulfur, cerium, and phosphorus over their thickness. In SEM images, ion exchanger grains in polyethylene (binder) and reinforcing fibers or holes left after their removal can be seen. Bright areas on the sulfur maps (Figure 1b,e,i) can be attributed to ion exchanger grains containing functional sulfonic acid groups. In the R_Ce membrane, cerium ions are predominantly distributed in the surface layer (the modified layer thickness does not exceed 20% of the membrane thickness (Figure 1c)). Since the cerium distribution in the modified membranes does not completely reproduce the sulfur distribution, it can be concluded that ceria is formed not only in ion exchanger grains, but also in large pores between the membrane components. A similar pattern is observed for the R_Ce_HP and R_Ce_NaP membranes (Figure 1d–k): Cerium ions are located on one side of each membrane, penetrating approximately 1/3 of the membrane thickness (about 150 μm). The layer with cerium ions is thicker in the R_Ce_HP and R_Ce_NaP membranes than in the R_Ce membrane, which may be due to a partial dissolution – precipitation of ceria in the membrane pores upon treatment with NaH_2_PO_4_ and especially H_3_PO_4_ solutions, which leads to diffusion of cerium ions and their redistribution over the membrane thickness. The distribution of phosphorus reproduces the distribution of cerium in the R_Ce_HP and R_Ce_NaP membranes. The R_Ce_NaP membrane shows the highest content of phosphorus. The Ce:P ratio in the R_Ce_HP and R_Ce_NaP samples is 9:1 and 3:1, respectively, suggesting only a partial modification of ceria by phosphate groups. It can be noticed that the phosphorus content is slightly higher in the near-surface layer of the R_Ce_HP membrane than in the total modified layer (Figure 1g). 

**Figure 2:**
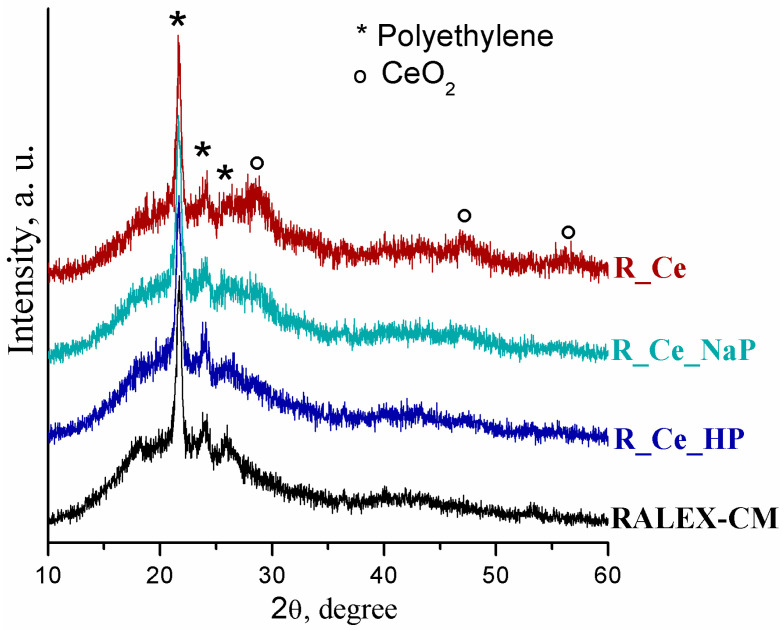
XRD patterns of the composite RALEX-CM membranes with ceria.

XRD patterns of the pristine and modified RALEX-CM membranes are shown in Figure 2. On the XRD pattern of the pristine RALEX-CM membrane, there are reflections of polyethylene at 2θ ≈ 22, 24 and 25° (PDF-2 database, #53-1859) against an amorphous halo. Additional lines at 2θ ≈ 28, 47 and 56° appear on the XRD pattern of the R_Ce membrane, which correspond to the reflections of cerium dioxide (PDF-2, #34-0394). It should be noted that metal oxides synthesized in the membrane pores are generally amorphous, even cerium oxide despite high crystallinity of its nanosized particles. To the best of our knowledge, the XRD pattern of the R_Ce membrane is the first example of XRD patterns of crystalline ceria in an ion exchange membrane. The significantly lower intensity of the ceria reflections on the XRD patterns of the R_Ce_NaP and R_Ce_HP membranes indicates the transformation of cerium oxide into amorphous cerium phosphate. In this case, the size of the cerium oxide particle decreases significantly, which leads to an additional broadening of its reflections and a decrease in their intensity. The XRD pattern of the R_Ce_HP membrane is almost the same as that of the RALEX-CM membrane.

Modification of ion exchange membranes by the in situ method occurs due to the formation of dopant particles in their pores with a size of 4–5 nm. Therefore, the size of formed particles usually does not exceed 4–5 nm, and they cannot be observed by SEM. TEM study of Nafion-117 membranes modified by CeO_2_ particles showed that the size of latter is about 2–3 nm [32]. CeO_2_ particles prepared out of membrane are larger, and their treatment with phosphoric acid leads to a slight decrease in their size [48]. Metal oxide particles embedded in membranes by in situ method are generally X-ray amorphous. In this work, reflections of CeO_2_ introduced into the ion exchange membrane were observed on X-ray diffraction patterns for the first time. This enables us to determine their coherent scattering region (crystallite size L) using the Scherrer Equation (5):

L = 0.9 λ/(β cosΘ_B_) (5)

where λ is the wavelength of the X-ray beam, and β represents the full width at half maximum of the reflection at the Bragg angle Θ_B_. The crystallite size of ceria in the R_Ce membrane is about 3 nm. However, the treatment of ceria with NaH_2_PO_4_ or H_3_PO_4_ leads to a chemical transformation on its surface with a decrease in the size of the CeO_2_ nucleus, as a result of which the CeO_2_ reflections on the X-ray diffraction patterns of the R_Ce_HP and R_Ce_NaP membranes disappear.

Figure S1 shows FTIR spectra of the composite membranes based on RALEX-CM membranes and ceria. Frequencies of both bending and stretching vibrations of ceria are below 400 cm^−1^, therefore these bands are not active in the IR region. In the R_Ce_HP and R_Ce_NaP membranes containing PO_4_-modified ceria, the bands corresponding to phosphate groups should be in the range of 1000–1100 cm^–1^ [49]. However, they overlap with the bands of sulfonic acid groups of membranes, and a low content of PO_4_-groups leads to the fact that the FTIR spectra of the pristine RALEX-CM membrane and modified membranes coincide.

With the introduction of cerium oxide into pores and channels of RALEX-CM, ion exchange capacity the R_Ce_HP membrane decreases (Table 1). This is due to the formation of salt bridges between the dopant and sulfonic acid groups of the RALEX-CM membrane. Apparently, the binding of hydrophilic sulfonic acid groups leads to an increase in the hydrophobicity of the membrane surface; the water contact angle increases from 88 to 100° (Table 1), which is close to that of the dry RALEX-CM membrane [50]. When the R-Ce membranes are treated with phosphoric acid or NaH_2_PO_4_ solutions, the ion exchange capacity increases, but its values are somewhat lower for the R_Ce_HP and R_Ce_NaP membranes than those of the pristine RALEX-CM membrane (Table 1). At the same time, due to a partial release of the membrane functional groups and an increase in its hydrophilicity, the water contact angle decreases. Its value are 83 and 81° for the R_Ce_HP and R_Ce_NaP membranes, respectively. However, the difference between these values should not be considered significant compared to the water contact angle of the pristine membrane.

In addition to a decrease in IEC and hydrophilicity of the R_Ce membrane, the formation of salt bridges upon the cerium oxide introduction leads to the “contraction” of the membrane pore walls with the displacement of water from pores and a decrease in water uptake. A partial destruction of salt bridges upon treatment with H_3_PO_4_ or NaH_2_PO_4_ solutions leads to an increase in the water uptake of the R_Ce_HP и R_Ce_NaP membranes compared to that of the R_Ce membrane (Table 1).

The introduction of ceria has little effect on cation transport numbers in the R_Ce membrane due to two competing processes. The formation of salt bridges reduces a negative charge of pore walls and the selectivity of membranes. However, the introduction of ceria nanoparticles leads to the displacement of an electrically neutral solution from the central part of membrane pores and a decrease in the co-ion concentration in them [51]. Treatment of the R_Ce membrane with phosphoric acid and NaH_2_PO_4_ solutions leads to a partial release of sulfonic acid groups and imparts a negative charge to the ceria surface due to the dissociation of phosphate groups [41]. As a result, the cation transport numbers increase up to 94.4–94.7% in the R_Ce_HP и R_Ce_NaP membranes (Table 1). It can be noted that similar results were obtained for the RALEX-CM membrane modified with hydrogen zirconium phosphate, for which the cation transport numbers reached 93%–97% [52].

**Figure 3:**
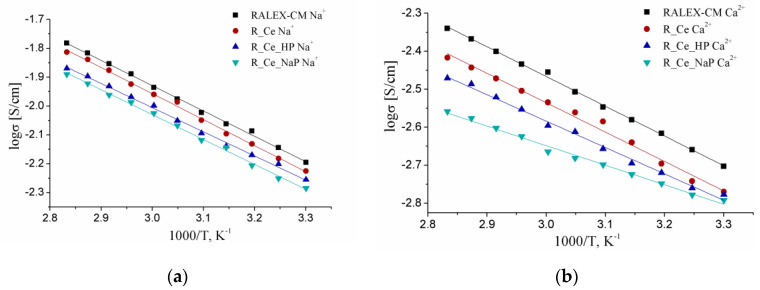
Temperature dependences of conductivity of the modified membranes in the Na^+^- (**a**) and Ca^2+^- (**b**) forms.

Due to reduced ion exchange capacity and water uptake, ionic conductivities of the modified membranes are somewhat lower than that of the pristine RALEX-CM membrane (Figure 3). Moreover, the conductivity of the modified membranes in the Ca^2+^ forms is significantly lower than that of the Na^+^ forms, which is associated with a lower mobility of doubly charged ions and a decrease in the water uptake of membranes in the Ca^2+^ form. This is also reflected in an increase in the activation energy of cation transfer with increasing cation charge (bonds between Ca^2+^ ions and water molecules/sulfonic acid groups are stronger) [51]. Conductivity of membranes in the Ca^2+^ forms decrease even more when cerium oxide is modified with PO_4_-groups, which have a high affinity for Ca^2+^ ions.

**Figure 4:**
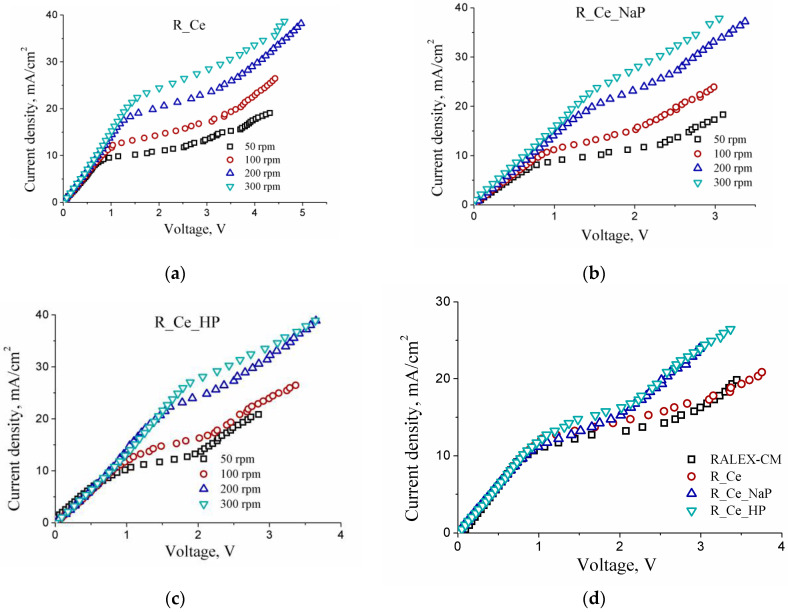
Current-voltage curves of the R_Ce (**a**), R_Ce_NaP (**b**) and R_Ce_HP (**c**) membranes in 0.03 mol-eq/L solution of NaCl and CaCl_2_ at different membrane disc rotation rates, and the comparison of CV curves obtained at a membrane disc rotation rate of 100 rpm (**d**).

Additional information about transport processes in ion exchange membranes can be obtained from CV characteristics of membranes. The CV curves were obtained in a mixed NaCl–CaCl_2_ solution at different rotation rates of the membrane disk (Figure 4). With increasing rotation rate, the limiting current increases, indicating an externally diffusive nature of the limiting current. The higher the electrolyte flow rate (the higher the membrane disk rotation rate), the smaller the diffusion layer thickness, and consequently, the higher the limiting current.

Figure 4d compares the current-voltage curves for the modified membranes at a membrane disk rotation rate of 100 rpm. The ohmic components (in the low ohmic region) for all membranes are similar for all membranes (Figure 4d). However, the limiting current density is higher for the modified membranes than for the pristine RALEX-CM membrane (Figure 4d, Table 1). This is most likely due to an increase in the carrier concentration due to the dissociation of –PO_4_M_X_ groups. An increase in the limiting current density will result in an improvement of the efficiency of water treatment by electrodialysis. Note also that the R_Ce_HP and R_Ce_NaP membranes pass into the over-limiting state at lower voltage (the width of the diffusion limited plateau region is narrower). Since the transport numbers t_+_ of the modified membranes are higher than those of the pristine membrane (Table 1), an increase in the limiting current density may be attributed to an enhancement of electro-convection on the surface of the modified membranes. Moreover, according to [45,53,54], the presence of phosphate groups in the modified membranes can catalyze water dissociation.

**Figure 5:**
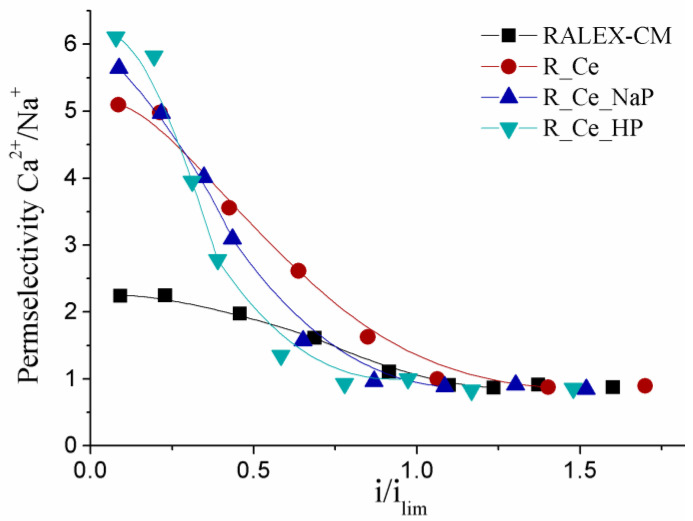
Dependence of selective permeability (P_Ca/Na_) of the modified membranes on the normalized current density (i/i_lim_) in 0.03 mol-eq/L solution of NaCl and CaCl_2_ at a membrane disk rotation rate of 100 rpm.

Differences in ionic mobilities of singly and doubly charged ions and the current-voltage characteristics of the modified membranes can be considered as their advantage for the separation of ions with different charges. Figure 5 shows the dependence of the permselectivity (P_Ca/Na_) for the modified membranes in a mixed CaCl_2_–NaCl solution. Modification of the membrane surface leads to a threefold increase in the selectivity to Ca^2+^ ions at low current densities. The highest increase in the selectivity to doubly charged ions was achieved for the R_Ce_HP membrane. However, the permselectivity of all membranes decreases with increasing current density. The difference in the separation of sodium and calcium ions is completely lost at the limiting current density. This is due to the fact that this process no longer depends on membrane characteristics and is determined only by diffusion coefficients and charges of ions in a solution, which correlates well with the conclusions made when analyzing the dependences of current-voltage profiles on membrane disk rotation rate.

To test the stability of the R_Ce_HP and R_Ce_NaP membranes, additional measurements were performed at a current density of 6 mA/cm^2^ (1/2 of the limiting current density) and a membrane disk rotation speed of 100 rpm for 48 h. After these tests, the selective permeability of these membranes remains almost the same (the deviation does not exceed 5% from the initial value), which indicates their high stability and efficiency in electrodialysis applications at low current densities.

The efficiency of this approach is worth comparing to those proposed in recent works. The layer-by-layer technique of the membrane surface modification with layers of polyelectrolytes, which provide the transfer of ions of opposite charge, is most often used to increase the selectivity of membranes to single charged ions [26,27,55,56,57]. Such mem-branes exhibit very high permselectivities, which are a thousand times more than those of pristine membranes, but at the same time their resistances increase hundreds of times and the limiting current decreases [58]. This significantly reduces the efficiency of ion separation. In this regard, approaches associated with the membrane surface modifica-tion with inorganic particles or a single layer of polyelectrolyte seem to be more preferable. Malik et al. reported a decrease in calcium transfer numbers from 89.0% to 73.3% after the membrane surface modification with polyaniline [59]. The surface modification of RALEX-CM membranes with hydrogen zirconium phosphate leads to a decrease in the selectivity to calcium ions over sodium ions to 0.7 [52]. Higher limiting current densities of the R_Ce_HP membranes compared to MK-40 membranes (their composition is similar to that of RALEX-CM membranes) with a thin Nafion layer [60] allow us to expect their high efficiency in electrodialysis.

## 4. Conclusions

Novel composite membranes based on RALEX-CM membranes and ceria were prepared. The proposed approach allows manufacturing membrane materials asymmetrically modified with cerium oxide, including cerium oxide with a surface functionalized with PO_4_-groups and provides guidance for synthesis of composite ion exchange membranes with improved selectivity. The modified membranes have lower ion exchange capacities and water uptakes than a pristine RALEX-CM membrane. However, their cation transport numbers increase, indicating an increase in the selectivity to cations. The composite membranes exhibit a threefold increase in the P_Ca/Na_ permselectivity at low current densities, which indicates the prospects for their use for the separation of singly and doubly charged ions in water treatment using electrodialysis.

## Figures and Tables

**Table 1:** Water uptake (ω(H_2_O)), ion exchange capacity, water contact angle (θc, from the modified side), cation transport numbers (t_+_), and limiting current density for the composite RALEX-CM membranes.

Membrane	ω(H_2_O), ±0.5%	IEC, ±0.05 mmol/g	θc, ±1°	t_+_, ±0.1%	Limiting Current Density, mA/cm^2^
RALEX-CM	31.3	1.34	88	93.8	11.13
R_Ce	24.8	0.98	100	93.9	11.97
R_Ce_HP	26.3	1.22	83	94.4	13.08
R_Ce_NaP	26.6	1.13	81	94.7	11.72

## Data Availability

The data presented in this study are available upon request from the corresponding author.

## Supplementary Materials

The following supporting information can be downloaded at: https://www.mdpi.com/article/10.3390/polym15030647/s1, Figure S1: FTIR spectra of the composite RALEX-CM membranes with ceria.
