# Supplementary figures and images for: Improvement of Selectivity of RALEX-CM Membranes via Modification by Ceria with a Functionalized Surface
Source: Polymers (Basel). 2023 Jan 27;15(3):647. doi: 10.3390/polym15030647 (PMC9919321; doi:10.3390/polym15030647)

## Supporting information

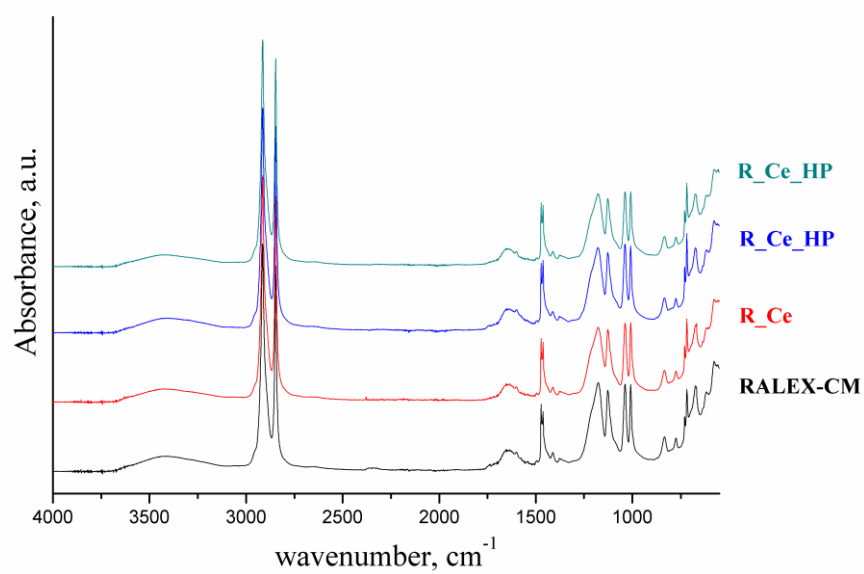

**Figure S1.** FTIR spectra of the composite RALEX-CM membranes with ceria.

Supplement: Supplementary file 1 [file polymers-15-00647-s001.zip › polymers-2157600-supplementary.pdf]
